# One-Year Follow-Up Study of Changes in Prostaglandin-Associated Periorbital Syndrome After Switch From Conventional Prostaglandin F2alfa to Omidenepag Isopropyl

**DOI:** 10.7759/cureus.10064

**Published:** 2020-08-27

**Authors:** Satomi Oogi, Shunsuke Nakakura, Etsuko Terao, Yasuko Fujisawa, Hitoshi Tabuchi, Yoshiaki Kiuchi

**Affiliations:** 1 Ophthalmology, Saneikai Tsukazaki Hospital, Himeji, JPN; 2 Ophthalmology, Hiroshima University, Hiroshima, JPN

**Keywords:** omidenepag isopropyl, prostaglandin ep2, glaucoma, prostaglandin f2α analogs, bimatoprost, prostaglandin-associated periorbital syndrome

## Abstract

Background

Cosmetic problems induced by conventional prostaglandin F2α (PGF2α) analogs are common. We prospectively evaluated the improvement of patients with prostaglandin-associated periorbital syndrome (PAPS) for whom the treatment regimen was switched from conventional PGF2α analogs to a new selective prostaglandin-EP2 agonist (i.e., omidenepag isopropyl).

Methods

We finally evaluated 12 patients with follow-up for one year who changed the therapy from conventional PGF2α drugs to omidenepag isopropyl. Digital facial images of the patients were captured prior to the initiation of therapy with omidenepag isopropyl and after approximately three, six, and 12 months. Three independent observers judged the recovery according to the five signs of PAPS - deepening of the upper eyelid sulcus (DUES), flattening of the lower eyelid bags, upper eyelid ptosis, ciliary hypertrichosis, and periorbital skin hyperpigmentation - by comparing images at baseline and each month.

Results

The mean age of patients (eight females; four males) was 61 years. The original PGF2α drugs were bimatoprost (N = 7), latanoprost (N = 3), travoprost (N = 1), and tafluprost (N = 1). The mean duration of treatment with PGF2α was 61 months. PAPS signs were evaluated in 11 patients after three months and in all 12 patients after six and 12 months. After three, six, and 12 months, DUES improved in five, six, and six patients, respectively; flattening of the lower eyelid bags improved in two, two, and three patients, respectively; upper eyelid ptosis improved in zero, one, and two patients, respectively; ciliary hypertrichosis improved in zero, one, and zero patients, respectively; and eyelid pigmentation improved in one, five, and three patients, respectively. Recovery of DUES was the most observed sign at ≤50%, whereas the recovery of ciliary hypertrichosis was the least sign at ≤8% at 12 months. All patients with improved DUES at one year had been receiving bimatoprost or travoprost.

Conclusions

Some PAPS signs improved after the administration of omidenepag isopropyl for one year. Our findings are useful for patients suffering from cosmetic problems induced by conventional PGF2α analogs.

## Introduction

Glaucoma is one of the major leading causes of blindness worldwide, and the reduction of intraocular pressure (IOP) is the only available evidence-based treatment that reduces the risk of visual field deterioration in this condition [1-2]. IOP-lowering medicated eyedrops, especially conventional prostaglandin F2α (PGF2α) analogs, have been popular as the first-line treatment for glaucoma. However, patients’ adherence or compliance plays a crucial role in treatment persistence. Adverse effects, such as “prostaglandin-associated periorbitopathy” (PAP), which causes cosmetic problems or disfigurement in periorbital changes, have recently been recognized by clinicians [3-7]. PAP is characterized by eight clinical features. Recently, Sarnoff and Gotkin proposed the term “prostaglandin-associated periorbital syndrome” (PAPS), which includes ciliary hypertrichosis and iris and periorbital skin hyperpigmentation in addition to PAP [8]. “PAPS” may account for all cosmetic side effects around the eyes that are caused by PGF2α.

In November 2018, omidenepag isopropyl 0.002% ophthalmic solution (EYBELIS®; Santen Pharmaceutical Co., Ltd., Osaka, Japan), a selective prostaglandin E2 receptor 2 agonist, with a non-prostaglandin structure, became available in Japan for the treatment of glaucoma and ocular hypertension. The IOP-lowering efficacy of omidenepag isopropyl is comparable to that of latanoprost 0.005% [9] and results in IOP reduction in non-/low-responder patients to latanoprost [10]. Recently, we reported the recovery of PAPS signs following a treatment switch from conventional PGF2α to omidenepag isopropyl during a six-month follow-up [11]. Some PAPS signs were improved during the six months, however, some were not [11]. Therefore, further long-term observation is needed to determine all the effects of omidenepag isopropyl on PAPs. The aim of this study was to prospectively reevaluate and reconfirm the recovery of patients who suffered from PAPS after switching the therapeutic regimen from conventional PGF2α to omidenepag for a one-year follow-up.

## Materials and methods

Patients and methods

This study was approved by the institutional review board of Saneikai Tsukazaki Hospital, Himeji, Japan, and performed according to the tenets of the Declaration of Helsinki [12].

We prospectively recruited patients who will change conventional PGF2α therapy to omidenepag isopropyl 0.002% therapy from November 2018 to July 2019 and followed up to July 2020. All patients had not undergone cataract surgery. We excluded patients who had a follow-up period of <12 months or who had lost a taken photograph more than two times or who discontinued the omidenepag isopropyl therapy. Finally, we evaluated 12 patients with open-angle glaucoma. The mean age of the 12 patients was 61 years (range: 45-75 years; eight females; four males). The mean duration of PGF2α use was 61 months (range: 24-115 months). The PGF2α drugs used previously were bimatoprost 0.03% (seven eyes), latanoprost 0.005% (three eyes), travoprost 0.004% (one eye), and tafluprost 0.0015% (one eye). Eleven patients were already included in a previous study [11].

Digital images of each patient’s face were captured prior to the initiation of therapy with omidenepag isopropyl and after approximately three, six, and 12 months. We used a digital single-lens camera (EX-ZR200 Exilim; Casio, Tokyo, Japan) with an auto flash mode. PAPS consists of 10 signs: deepening of the upper eyelid sulcus (DUES), flattening of the lower eyelid bags (FLEB), upper eyelid ptosis, involution dermatochalasis, orbital fat atrophy, mild enophthalmos, inferior scleral show, a tight orbit, ciliary hypertrichosis, and hyperpigmentation of the iris and periorbital skin. Of these signs, five were evaluated by three independent observers for improvement: DUES, FLEB, upper eyelid ptosis, ciliary hypertrichosis, and periorbital skin hyperpigmentation (the other signs are difficult to judge) [6,11]. Images were presented in Microsoft PowerPoint (Office 365; Microsoft Corporation, Redmond, WA) using a 16:9 screen in a pair of baseline images and those obtained at three or six and 12 months. Each observer independently judged the signs in the 21.5-inch monitor by expanding the PowerPoint to the whole screen [11]. If necessary, each observer further expanded the images. If both eyes of a patient were being treated with omidenepag isopropyl, the observers judged the appearance of the right eye.

To validate and maintain the fairness of our previous judging method, one observer was changed from the previous study (S.N. to S.O.). Each observer (E. T., Y.S., and S.O.) scored the judgment in a scoring sheet to check for improvement by comparing the baseline images with those captured at three, six, and 12 months (○). In the absence of improvement, the score was (×). After scoring, we collected the sheets and proceeded toward the final judgment. We used strict criteria for our judgment. If all three observers rated a sign as improved (○), the result was defined as “recovery.” Therefore, in case of a split decision (two observers scored (○) and one scored (×)) the result was judged as “no improvement” (×). Any discussions among the observers for the score were not used as a previous report [11].

Additionally, we measured the IOP using a Goldmann applanation tonometer at approximately three months prior to initiating therapy with omidenepag isopropyl, the day of treatment initiation, and at three, six, and 12 months later.

Statistical analysis

Statistical analyses were performed using the Statcel 3 (OMS Publishing Ltd., Tokyo, Japan) software. We used Fisher’s exact test to evaluate whether the previous drugs affected the incidence of recovery of PAPS signs at three, six, and 12 months. We divided the patients into two groups: previous users of bimatoprost or travoprost (total: N = 8) and previous users of latanoprost or tafluprost (total: N = 4). By objective evaluation, a previous study showed that the former group has a high incidence of DUES (60% for bimatoprost and 50% for travoprost versus 24% for latanoprost and 18% for tafluprost) [13].

Baseline IOP was set as the mean of IOP values at approximately three months prior to initiating omidenepag isopropyl and the day of initiation. The change in IOP throughout the period was evaluated using a one-way analysis of variance. P-values < 0.05 denoted statistically significant differences.

## Results

Table 1 shows all the judgment data. PAPS signs were evaluated in 11 patients after three months and in all 12 patients after six and 12 months. All patients were evaluated for 12 months, except for one patient who was not photographed at three months. The data of patients are summarized in Table 2. In particular, DUES improved in five patients at three months and six patients at both six and 12 months; FLEB improved in two patients at three and six months, and in three patients at 12 months; upper eyelid ptosis improved in zero patients at three months, one patient at six months, and two patients at 12 months; ciliary hypertrichosis improved in one patient only at three months; and periorbital skin hyperpigmentation improved in one patient at three months, in five patients at six months, and three patients at 12 months. Thus, the recovery of DUES was most pronounced at 12 months. Among six patients with improved DUES at 12 months, five had previously received bimatoprost and one had been treated with travoprost.

**Table 1:** Summary of the improvement of PAPS signs (compared with baseline) PAPS: prostaglandin-associated periorbital syndrome; DUES: deepening of the upper eyelid sulcus; FLEB: flattening of the lower eyelid bags

	Number of improved patients compared with baseline		
	At 3 months (N = 11)	At 6 months (N = 12)	At 12 months (N = 12)
DUES	5	6	6
FLEB	2	2	3
Upper eyelid ptosis	0	1	2
Ciliary hypertrichosis	0	1	0
Periorbital skin hyperpigmentation	1	5	3

**Table 2:** Patient demographics and the final judgment data for improvement of PAPS signs ○: All three evaluators unanimously judged the sign as an improvement; ×: the sign was judged as not improved; F: female; M: male; L: left; R: right; PG: prostaglandin; DUES: deepening of the upper eyelid sulcus; FLEB: flattening of the lower eyelid bags; POAG: primary open-angle glaucoma; NTG: normal-tension glaucoma

Patient number	Sex	Age (years)	Diagnosis	Preceded PG	Duration of PG (months)	Eye	At 3 months					At 6 months					At 12 months				
							DUES	FLEB	Upper eyelid ptosis	Ciliary hypertrichosis	Periorbital skin hyperpigmentation	DUES	FLEB	Upper eyelid ptosis	Ciliary hypertrichosis	Periorbital skin hyperpigmentation	DUES	FLEB	Upper eyelid ptosis	Ciliary hypertrichosis	Periorbital skin hyperpigmentation
1	F	54	POAG	Bimatoprost	48	L	×	×	×	×	×	〇	×	〇	×	×	〇	〇	〇	×	×
2	F	50	POAG	Latanoprost	67	R	○	×	×	×	×	×	×	×	×	×	×	×	×	×	×
3	F	45	NTG	Tafluprost	74	R	×	×	×	×	×	×	×	×	×	×	×	×	×	×	×
4	M	66	NTG	Travoprost	111	R	〇	×	×	×	×	〇	×	×	×	〇	〇	×	×	×	×
5	F	61	NTG	Bimatoprost	89	R	〇	×	×	×	×	〇	×	×	×	〇	×	×	〇	×	×
6	F	68	POAG	Bimatoprost	44	R	×	×	×	×	〇	×	×	×	×	×	×	〇	×	×	×
7	M	70	POAG	Bimatoprost	115	R	〇	〇	×	×	×	〇	〇	×	×	×	〇	×	×	×	〇
8	F	57	POAG	Bimatoprost	57	R	×	×	×	×	×	×	×	×	〇	〇	〇	×	×	×	×
9	F	67	POAG	Latanoprost	36	R	×	×	×	×	×	×	×	×	×	×	×	×	×	×	×
10	M	61	POAG	Latanoprost	24	R	No images					×	×	×	×	〇	×	×	×	×	×
11	M	75	POAG	Bimatoprost	25	L	〇	×	×	×	×	〇	×	×	×	×	〇	×	×	×	〇
12	F	66	POAG	Bimatoprost	44	L	×	〇	×	×	×	〇	〇	×	×	〇	〇	〇	×	×	〇

**Figure 1:**
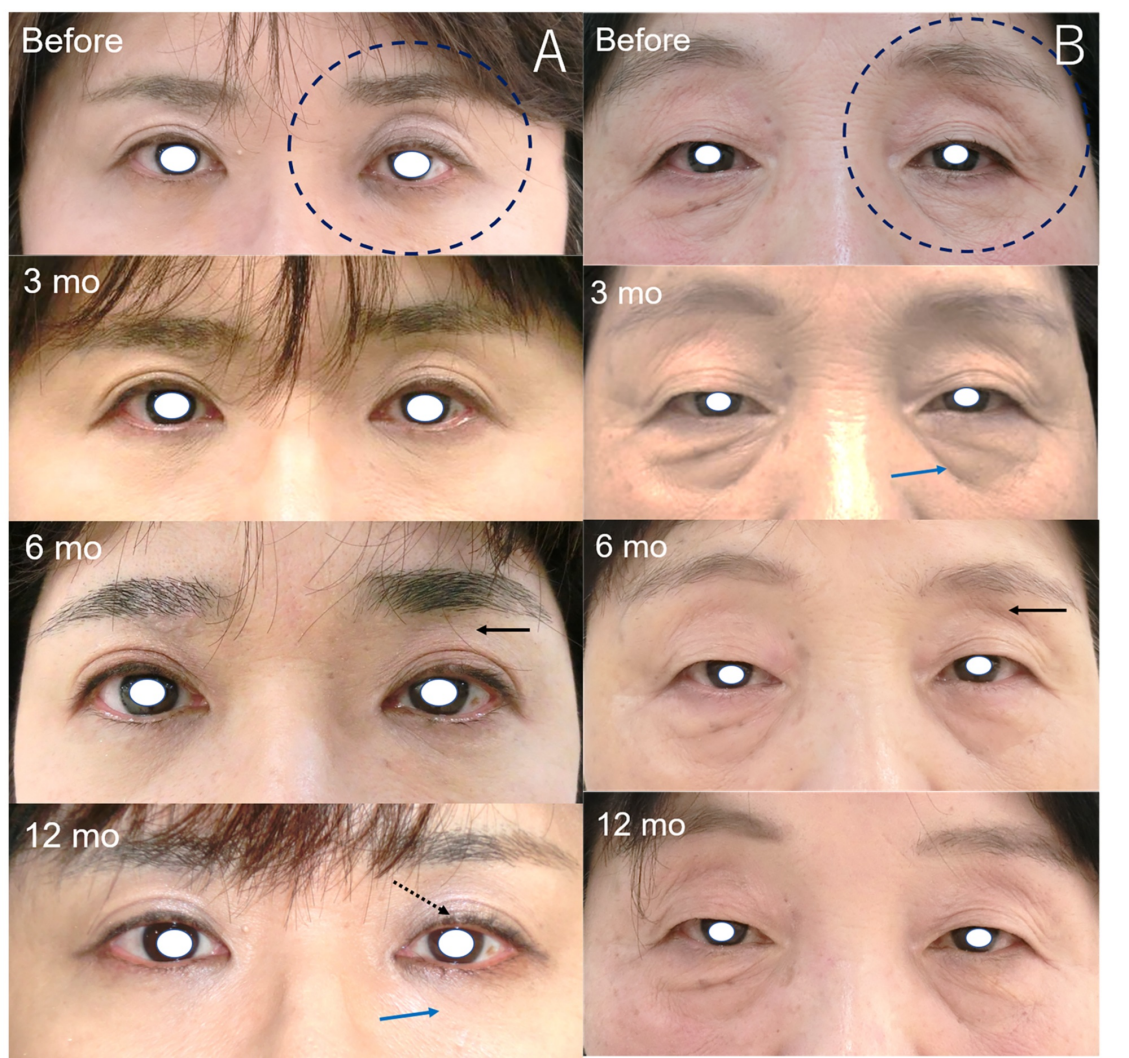
Images of the patients showing improvements in some PAPS signs a: Patient number 1. A 54-year-old female who had previously used bimatoprost for four years. The judged eye was the left eye (black dot circle). DUES (black arrow) and upper eyelid ptosis (black dot arrow) were judged as improved at six and 12 months, and FLEB (blue arrow) was judged as improved at 12 months after switching to therapy with omidenepag isopropyl. b: Patient number 12. A 66-year-old female who previously used bimatoprost for 44 months. The judged eye was the left eye (black dot circle). DUES (black arrow) and periorbital skin hyperpigmentation were judged as improved at six and 12 months, and FLEB (blue arrow) was judged as improved at three, six, and 12 months. DUES, deepening of the upper eyelid sulcus; FLEB; flattening of the lower eyelid bags; PAPS, prostaglandin-associated periorbital syndrome

**Figure 2:**
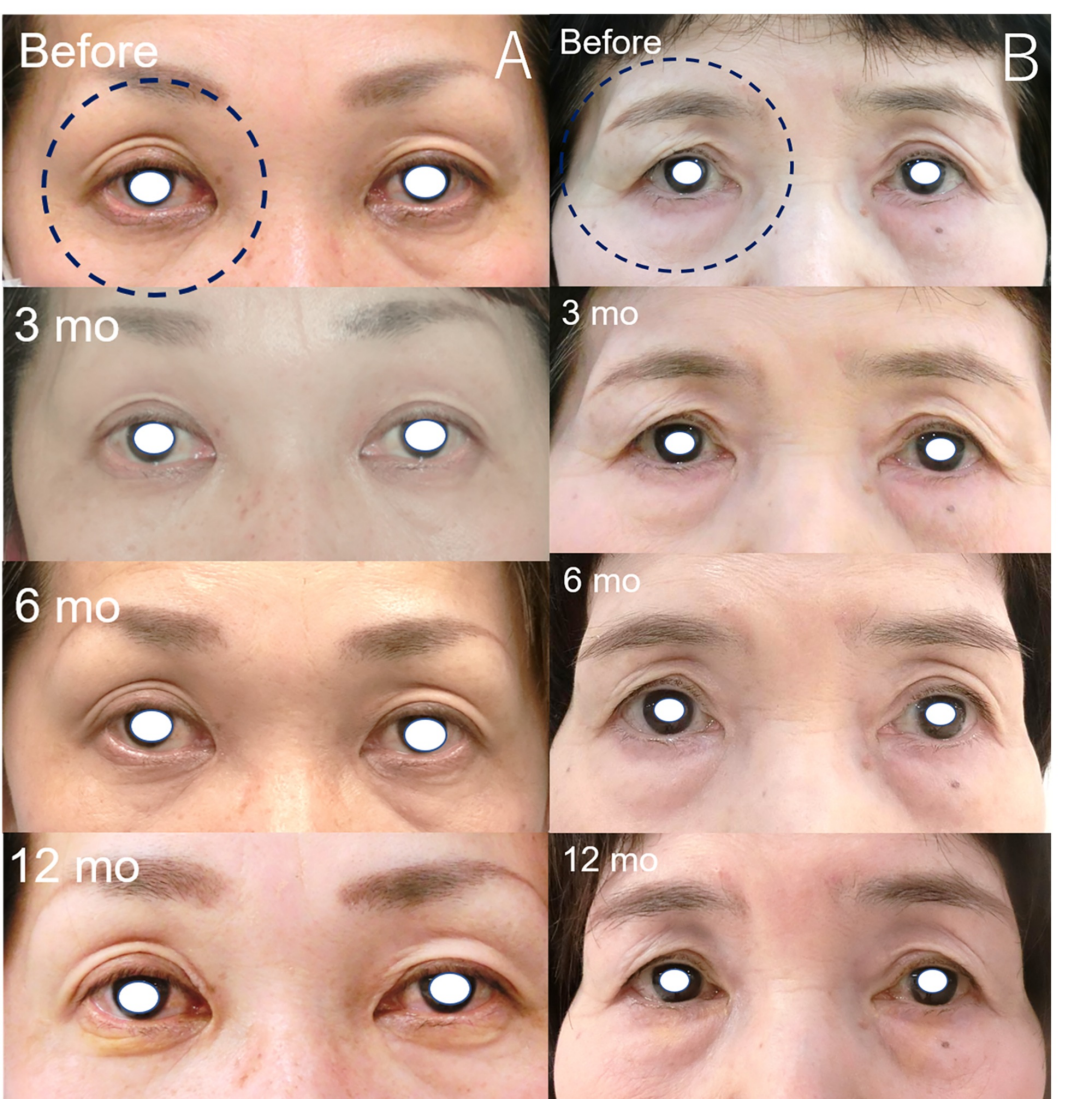
Images of patients not showing marked improvement in some PAPS signs a: Patient number 2. A 50-year-old female who previously used latanoprost for 67 months. The judged eye was the right eye (black dot circle). DUES was judged as improved at three months; however, the judgment was changed at six and 12 months after switching to therapy with omidenepag isopropyl. The other signs were not improved. b: Patient number 9. A 67-year-old female who previously used latanoprost for 67 months. There were no apparent changes observed after switching to therapy with omidenepag isopropyl. DUES, deepening of the upper eyelid sulcus; PAPS, prostaglandin-associated periorbital syndrome

Two previous users of bimatoprost (numbers 1 and 12) who had relatively improved PAPS signs (Figure 1) showed improvements in DUES and FLEB at 12 months. Meanwhile, two previous users of latanoprost (numbers 2 and 9) did not show marked changes in PAPS signs (Figure 2). We obtained additional written informed consent from the patients shown in the figures for publication of their facial photographs.

We investigated the association between the previous drugs and the recovery of PAPS signs at each month. The results showed that improvement of DUES was more frequently observed in previous users of bimatoprost or travoprost than in previous users of latanoprost or tafluprost at six and 12 months (P = 0.030, by Fisher’s exact test). There was no significant difference found in any of the other signs (all P-values > 0.2).

Mean IOPs were 16.5±1.5 mmHg, 15.4±1.9 mmHg, 15.5±1.7 mmHg, and 15.9±1.8 mmHg at baseline, and three, six, and 12 months, respectively. There was no significant change in IOP observed during the follow-up period (P = 0.394 by one-way analysis of variance).

## Discussion

We confirmed the recovery of some PAPS signs after switching from conventional PGF2α to omidenepag isopropyl for a one-year prospective follow-up. The recovery of DUES was the most frequently observed (≤50%), especially in previous users of bimatoprost and travoprost. Additionally, IOPs were maintained for 12 months. Among PAPS signs, DUES, FLEB, involution dermatochalasis, orbital fat atrophy, and mild enophthalmos are thought to be the result of PGF2α-induced fat atrophy [3,6,14], which is caused by the inhibition of adipocyte differentiation [15] and adipogenesis [16]. Recently, it was shown that prostaglandin F agonists inhibit adipogenesis by stimulating the prostaglandin F receptor in 3T3-L1 cells [17]. However, omidenepag isopropyl did not affect the differentiation of adipocytes [18]. Therefore, in our results, the recovery of DUES and FLEB were often observed. Regarding ciliary hypertrichosis, there was almost no apparent change observed over the 12 months. It takes approximately eight weeks for eyelashes to grow back after being pulled out [19]. The mechanisms of ciliary hypertrichosis induced by PGF2α drugs are thought to be the dysregulation of the eyelash growth rate and hair growth cycle [19]. Therefore, the effect of PGF2α drugs on the eyelash growth rate and hair growth cycle may last for 12 months because omidenepag isopropyl does not affect eyelash growth in mice, whereas bimatoprost does [20]. The resolution of periorbital skin hyperpigmentation in patients using bimatoprost required between three and 12 months (average: 205 days) after discontinuing bimatoprost; a longer duration of use was linked to a longer period of resolution [21]. Therefore, a gradual resolution is observed. There are no reports on the recovery from upper eyelid ptosis after discontinuation or switching of PGF2α. However, we suspect that patient number 1 experienced marked improvement (Figure 1a).

There were some limitations to this study. Firstly, the degree and incidence of PAPS signs at baseline in our study may differ for each PGF2α analog [4,6]. Therefore, patients who use bimatoprost or travoprost may have apparent PAPS at baseline versus those who use latanoprost or tafluprost. Patients who used latanoprost or tafluprost may have no sign at baseline. Unfortunately, we did not have photographs before the instillation of PGF2α because most patients were already treated before being referred to our hospital.

**Figure 3:**
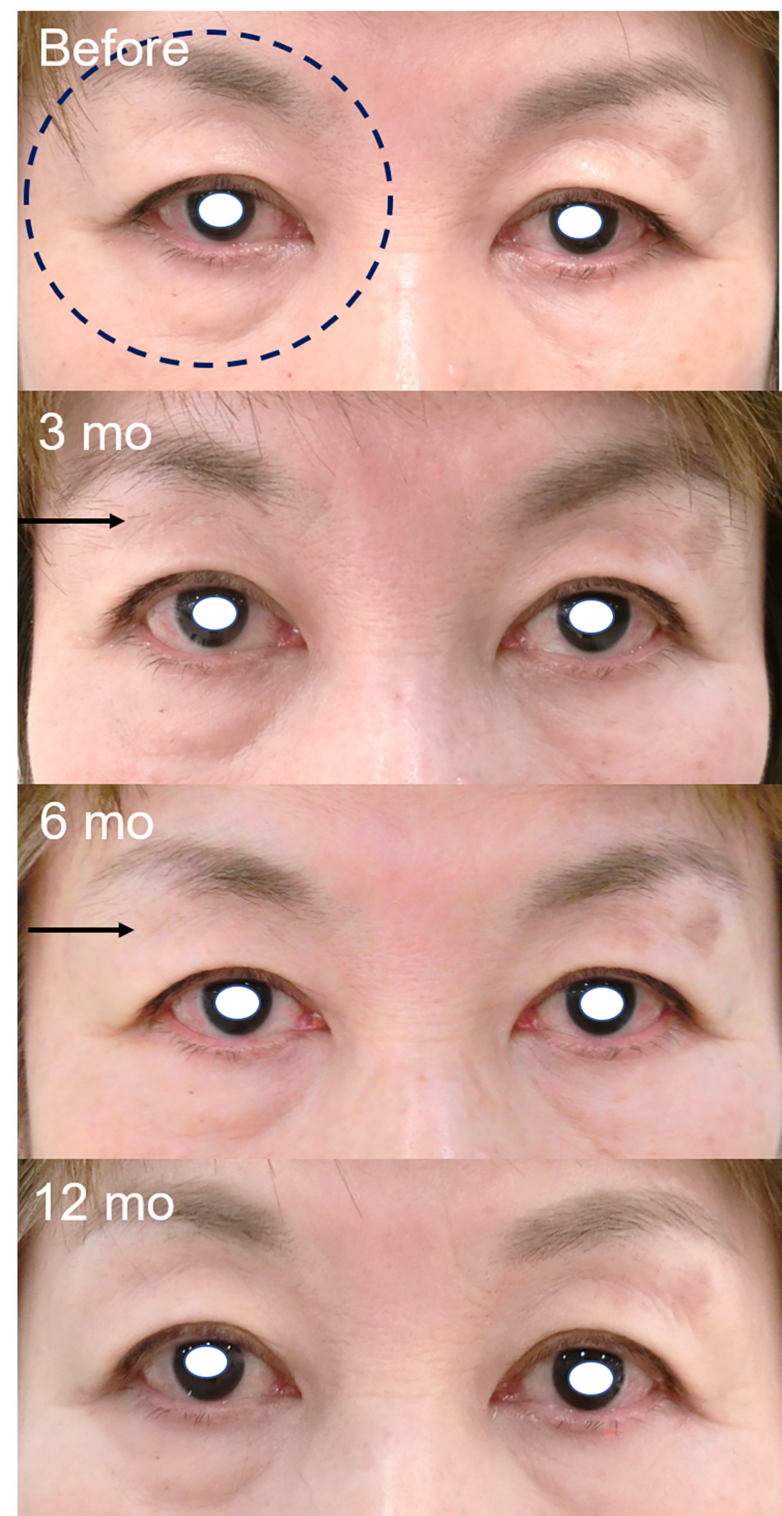
Patient for whom the judgment was changed at 12 months due to the condition under which the images were captured A 61-year-old female who previously used bimatoprost for 89 months. DUES (black arrow) was judged as improved at three and six months. However, at 12 months, DUES was judged as not improved, whereas upper eyelid ptosis was judged as improved. This was probably due to the effect of the wide-opening eye phenomenon [22]. Wide-opening eyes may cause the recovery of upper eyelid ptosis and induce a DUES-like appearance. DUES: deepening of the upper eyelid sulcus

Secondly, it was very difficult to unify the image conditions in clinical practice; the photographer, background, and patient’s facial expression may differ. Therefore, some images have slightly different color tones, affecting the judgment of periorbital skin hyperpigmentation. Additionally, we previously reported that wide-opening eyes would cause a DUES-like appearance [22]. Figure 3 shows that for patient number 5, the judgment of DUES was changed from three and six months (judged as improved) to 12 months (not improved) due to the wide-opening eye phenomenon [22]. Her wide-opening eyes may have decreased the upper eyelid ptosis and led to a DUES-like appearance. Thirdly, in this study, an observer from the previous study was replaced, and all patients were judged again to reconfirm the recovery of PAPS signs. Therefore, some results were different from those of the previous study. In our previous six months follow-up study, DUES improved in three patients, FLEB improved two patients, upper eyelid ptosis improved in zero patients, ciliary hypertrichosis improved in two patients, and periorbital skin hyperpigmentation improved in eight patients [11]. It was also better to take questionnaires about cosmetic changes before and after switching to omidenepag. Further long-term observation is required to determine all the effects of omidenepag isopropyl on PAPS.

## Conclusions

Some PAPS signs, particularly in DUES, FLEB, and periorbital skin hyperpigmentation, improved after the initiation of omidenepag isopropyl for a one-year follow-up. Our findings will be useful for alleviating cosmetic problems in patients who already suffer from PAPS and for preventing their occurrence in patients using newly prescribed antiglaucoma eye drops.
